# Identification and characterization of stable QTLs for vascular bundle number at the panicle neck in rice (*Oryza sativa* L.)

**DOI:** 10.1270/jsbbs.23013

**Published:** 2023-09-09

**Authors:** Ha Thi Le Nguyen, Shizuka Suetsugu, Yuna Nakamura, Zita Demeter, Shao-Hui Zheng, Daisuke Fujita

**Affiliations:** 1 The United Graduate School of Agricultural Sciences, Kagoshima University, 1-21-24 Korimoto, Kagoshima 890-8580, Japan; 2 Forest Science Institute of South Vietnam, 1 Pham Van Hai, Tan Binh District, Ho Chi Minh City, Viet Nam; 3 Faculty of Agriculture, Saga University, 1 Honjo-machi, Saga 840-8502, Japan

**Keywords:** rice, vascular bundle, stable QTLs, CSSLs, RILs, pyramided lines

## Abstract

A large vascular bundle number (VBN) in the panicle neck in rice (*Oryza sativa* L.) is related to the ability to transport assimilates from stem and leaf to reproductive organs during seed maturation. Several quantitative trait loci (QTLs) for VBN have been identified by using segregating populations derived from a cross between *indica* and *japonica* rice cultivars. However, the detailed location, effect, and interaction of QTLs for VBN were not understood well. Here, to elucidate the genetic basis of VBN, we identified three stable QTLs for VBN—*qVBN5*, *qVBN6* and *qVBN11*—by using 71 recombinant inbred lines derived from a cross between *indica* ‘IR24’ and *japonica* ‘Asominori’. We confirmed their positions and characterized their effects by using chromosome segment substitution lines (CSSLs) with an ‘IR24’ genetic background. *qVBN6* had the most substantial effect on VBN, followed by *qVBN11* and *qVBN5*. We developed pyramided lines carrying two QTLs for VBN to estimate their interaction. The combination of *qVBN6* and *qVBN11* accumulated VBN negatively in the pyramided lines owing to the independent actions of each QTL. The QTLs detected for VBN will enhance our understanding of genetic mechanisms of VBN and can be used in rice breeding.

## Introduction

Rice (*Oryza sativa* L.), an essential staple crop for more than half of the world’s population, is grown mainly in East, Southeast, and South Asia. Owing to the continuously increasing population size and loss of arable land resulting from land degradation and salinization of irrigated areas, increasing rice production per unit area is a major goal of breeders (Khush 1997). Grain yield is determined by four yield components: spikelet number per panicle, panicle number per plant, grain weight, and grain fertility. To increase yield potential, increasing the source size, sink size, and translocation capability are crucial (Donald 1968, Lee and Chae 2000). The vascular system plays an important role in transporting photosynthesis products from source to sink, and its transport capacity influences grain yield (Cui *et al.* 2003, Fukuyama *et al.* 1999). In the development of hybrid rice, a small vascular bundle number (VBN) in the panicle neck restricts grain filling by limiting the transport of assimilates to the sink (Peng *et al.* 1999, Xu *et al.* 2005).

Vascular bundles interconnect all parts of a plant and are responsible for transporting photosynthesis products, minerals, and water around (Lucas *et al.* 2013). Rice has two types of vascular bundles: small ones in the rudimentary glumes of spikelets, and large ones that play a major role in nutrient uptake and ripening rate, which in turn affect grain weight (Chaudhry and Nagato 1970). Wide genetic diversity among rice cultivars underlies the number of large vascular bundles in the panicle neck; the VBN of *indica* rice tends to be greater than that of *japonica* rice (Fukuyama and Takayama 1995, Fukuyama *et al.* 1999, Liu *et al.* 2016, Zhai *et al.* 2018); and the ratio of VBN to primary branch number differs between them (Fukuyama and Takayama 1995, Fukuyama *et al.* 1999).

VBN is influenced mainly by inherited factors but also by environmental factors such as nitrogen availability and plant density. Through the use of segregating populations such as recombinant inbred lines (RILs) and double haploid lines (DHs), genetic factors that control VBN in the panicle neck of *O. sativa* have been detected as quantitative trait loci (QTLs) (Bai *et al.* 2012, Cui *et al.* 2003, Sasahara *et al.* 1999, Zhang *et al.* 2002); and genome-wide association studies (GWAS) have identified QTLs for VBN (Liao *et al.* 2021, Zhai *et al.* 2018). Some genes underlying QTLs for VBN have been isolated to their chromosomal regions and cloned, and include *ABERRANT PANICLE ORGANIZATION 1* (*APO1*) on chromosome (Chr.) 6 (Terao *et al.* 2010), *NARROW LEAF 1* (*NAL1*) on Chr. 4 (Fujita *et al.* 2013, Qi *et al.* 2008), and *LVB9/DENSE AND ERECT PANICLE 1* (*DEP1*) on Chr. 9 (Fei *et al.* 2019).

In this study, RILs derived from a cross between *indica* rice ‘IR24’ and *japonica* rice ‘Asominori’ were used to elucidate the genetic basis of VBN. Chromosome segment substitution lines (CSSLs) can be used for developing materials for confirming QTL and for evaluating QTL epistasis through pyramiding (Yamamoto *et al.* 2009). Each detected QTL was confirmed in CSSLs carrying ‘Asominori’ chromosomal segments in the ‘IR24’ genetic background. QTL analysis of VBN was conducted using RILs, and each QTL’s phenotypic effects were verified and validated using CSSLs. To understand interaction of QTLs, the effects of pyramiding pair of the QTLs were evaluated.

## Materials and Methods

### Plant materials

For QTL analysis in 2016, 2017, and 2018, we used 71 RILs derived from a cross between ‘Asominori’ and ‘IR24’ followed by the single-seed-descent method (Tsunematsu *et al.* 1996). For evaluating the effect of QTLs in 2018 and 2019, we used three CSSLs with the ‘IR24’ genetic background—IAS30, IAS39, and IAS14—carrying target substitution chromosomal segments of ‘Asominori’ (Kubo *et al.* 2002). For QTL confirmation in 2019, we used three F_2_ populations derived from a cross between each of those CSSLs and ‘IR24’ (168 individuals each). To characterize the gene interactions among the detected QTLs, we developed and characterized pyramided lines (PYLs) each carrying two QTLs in the ‘IR24’ genetic background: we developed three F_1_ plants from crosses between IAS30 (carrying *qVBN5*) and IAS39 (carrying *qVBN6*), IAS30 and IAS14 (carrying *qVBN11*), and IAS39 and IAS14. These F_1_ plants were self-pollinated to develop F_2_ populations of 96 plants each. PYLs were selected from each F_2_ population by marker-assisted selection: PYLs 1–4 from IAS30/IAS14, PYLs 5–7 from IAS39/IAS14, and PYLs 8–10 from IAS30/IAS39. These 10 PYLs were self-pollinated, and the effects of pyramiding were evaluated in 24 plants of each F_3_ line in 2020. For evaluating the effect of QTLs in 2017, 2018 and 2019, we used three CSSLs with the ‘Asominori’ genetic background—AIS38, AIS49, and AIS76—carrying target substitution chromosomal segments of ‘IR24’ (Kubo *et al.* 2002).

### Plant growth condition and evaluation of VBN

Plants were grown in a paddy field of Saga University (33°14ʹ32ʺN 130°17ʹ24ʺE). At 28 days after sowing, seedlings were transplanted at one plant per hill with 20 cm between hills and 25 cm between rows. Inorganic fertilizer was applied at 40 kg/ha N, 17.5 kg/ha P, and 33 kg/ha K. At 2 to 3 weeks after heading, the tallest panicles among the individual plants were collected for counting of VBN in the panicle neck. Fresh peduncles were sliced at about 1 cm below the panicle base node, and the large vascular bundles were counted under a microscope, in 5 stems each of RILs and PYLs, 10 of CSSLs, and every plant in the F_2_ populations.

### Extracting DNA and genotyping

Approximately 2–4 cm of leaves was collected directly and freeze-dried for 48 h. Total DNA was extracted by the potassium acetate method (Dellaporta *et al.* 1983). Polymorphic SSR markers (McCouch *et al.* 2002) and indel markers (Yonemaru *et al.* 2015) were used for genotyping of segregating populations; these co-dominant markers distinguish between the two parental lines. Polymerase chain reaction (PCR) was performed using GoTaq master mix (Promega) at 96°C for 5 min; 35 cycles of 30 s at 96°C, 30 s at 55°C, and 30 s at 72°C; and a final at 25°C for 1 min. The amplified PCR products were run in 4% agarose gel at 200 V with 0.5 μg/mL ethidium bromide in 0.5 × TBE buffer for 60–120 min.

### DNA markers

QTLs in RILs were found using the genotyping data from RFLP markers each year (Tsunematsu *et al.* 1996). QTL analysis for VBN in each population used SSR markers located around the substituted chromosome locations in the CSSLs. We selected 15 SSR/indel markers on Chr. 5 around RFLP marker interval C128–R2117 (22.6–23.4 Mbp) for genotyping the IR24/IAS30 F_2_ population; 7 markers on Chr. 6 around C962–Ky11 (~28.6 Mbp) for genotyping the IR24/IAS39 F_2_; and 12 markers on Chr. 11 around R2918–C3029A (~2.2–2.4 Mbp) for genotyping the IR24/IAS14 F_2_ (Supplemental Table 1).

### QTL analysis

We performed composite interval mapping in Windows QTL Cartographer v. 2.5 software (Wang *et al.* 2012) to identify QTLs for VBN. The critical threshold values of logarithm of odds (LOD) that were calculated by 1000 permutation tests with significant level at *P* < 0.05 on each population were 3.15 in 2016, 3.23 in 2017 and 3.10 in 2018 for RILs and 2.07 for IAS30/IR24 F_2_ population, 1.67 for IAS14/IR24 F_2_ population and 1.89 for IAS39/IR24 F_2_ population.

### Statistical analysis

One-way ANOVA assessed the phenotypic differences among parents, CSSLs carrying single, and PYLs carrying paired QTLs. Tukey Kramer’s test was performed for multiple comparison of VBN among parents, CSSLs, and PYLs and Dunnett’s test was used to compare VBN between ‘Asominori’ and CSSLs using the R software, version 3.5.2.

## Results

### Identification of QTLs for VBN in RILs

**Fig. 1.**
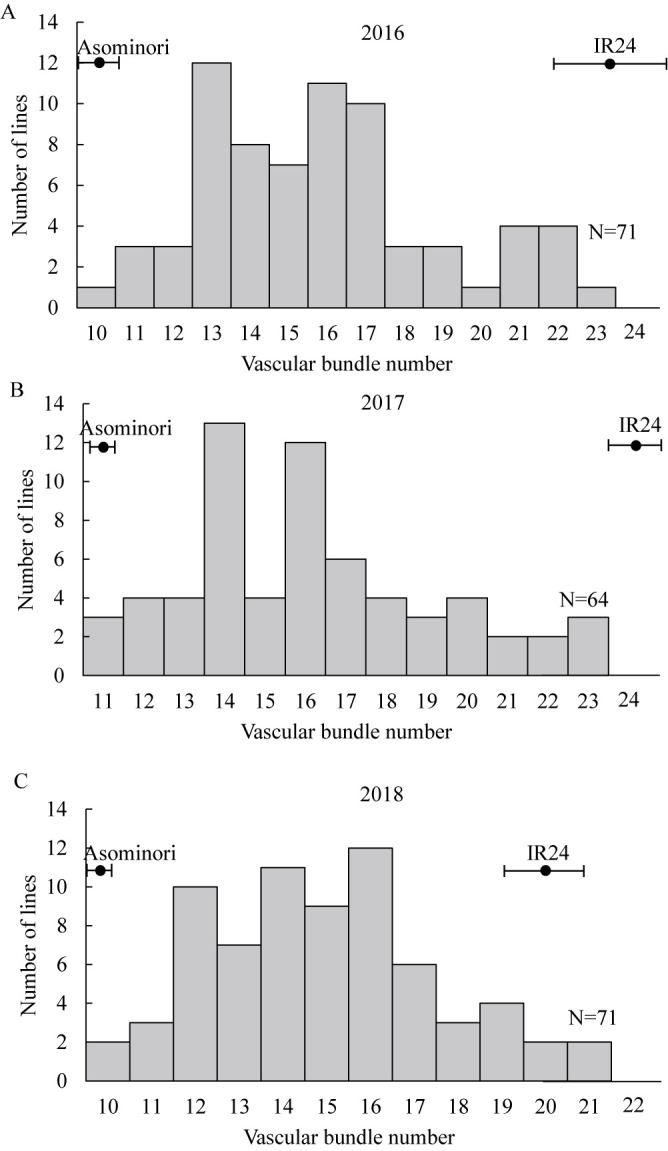
Frequency distributions of vascular bundle number in panicle neck in RILs in (A) 2016, (B) 2017, (C) 2018. Bars indicate means in parents with standard deviation.

The VBN of ‘Asominori’ was 10.3–11.8 in all 3 years, and that of ‘IR24’ was 20.4–24.8 (Fig. 1). Those of the RILs were 10–23 in 2016, 11–23 in 2017, and 10–21 in 2018. The continuous frequency distributions of VBN in the RILs imply that multiple genetic factors control VBN.

Four QTLs for VBN were detected: *qVBN5* and *qVBN6* in 2016, 2017, and 2018; *qVBN11* in 2016 and 2017; and *qVBN4* in 2018 (Table 1). *qVBN5* had PVE of 10.3%–28.6%, *qVBN6* of 13.6%–18.3%, *qVBN11* of 11.8%–24.0%, and *qVBN4* of 10.4%. The ‘Asominori’ alleles at *qVBN5*, *qVBN6*, and *qVBN11* decreased VBN, whereas that at *qVBN4* increased VBN.

### Validation of single QTL effects on VBN in ‘IR24’ genetic background

**Fig. 2.**
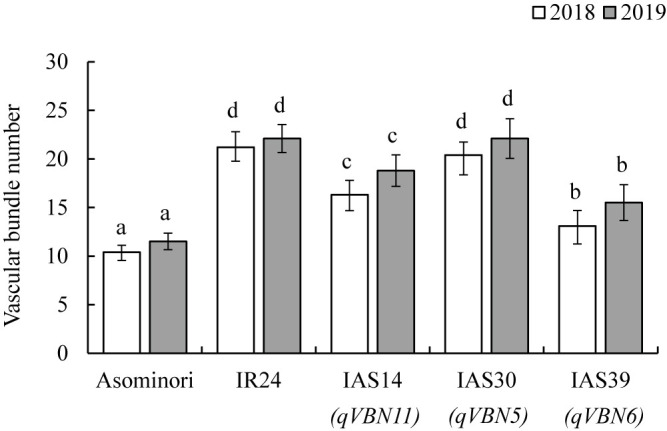
Effect of each QTL for vascular bundle number in panicle neck in parents and CSSLs. Bars indicate standard deviation. Bars with the same letter are not significantly different between genotypes by Tukey–Kramer multiple comparison test (*P* < 0.05).

In 2018, VBN of IAS39 (carrying *qVBN6*) was 13.1, and that of IAS14 (carrying *qVBN11*) was 16.3 (Fig. 2), significantly lower than that of ‘IR24’ (21.2). On the other hand, VBN of IAS30 (carrying *qVBN5*) was 20.4, similar to that of ‘IR24’. The ‘Asominori’ alleles at *qVBN6* and *qVBN11* decreased VBN by 8.1 in IAS39 and by 4.9 in IAS14. In 2019, VBN of IAS39 was 15.5 and that of IAS14 was 18.8, significantly lower than that of ‘IR24’ (22.1). On the other hand, VBN of IAS30 was 22.1, close to that of ‘IR24’. The ‘Asominori’ alleles at *qVBN6* and *qVBN11* decreased VBN by 6.6 in IAS39 and by 3.3 in IAS14. The effects of the three QTLs were consistent between years, implying minor environmental influence on VBN. These results indicate that the ‘Asominori’ alleles at *qVBN6* and *qVBN11* stably decreased VBN and validate their effects in the ‘IR24’ genetic background.

Along with evaluating of ‘Asominori’ allele in ‘IR24’ genetic background, we used three CSSLs with the ‘Asominori’ genetic background—AIS38 (carrying *qVBN5*), AIS49 (carrying *qVBN6*), and AIS76 (carrying *qVBN11*)—carrying target substitution chromosomal segments of ‘IR24’. The VBN of these CSSLs showed slightly incerease in VBN as compare to that of ‘Asominori’ (Supplemental Table 2). For confirmation of these QTLs, the detections of QTLs for VBN under ‘Asominori’ genetic background were difficult because of small difference of VBN between AIS and ‘Asominori’. Therefore, we performed following experiments under ‘IR24’ genetic background to confirm these detected QTL for VBN in RILs.

### Confirmation of QTLs for VBN using CSSLs

**Fig. 3.**
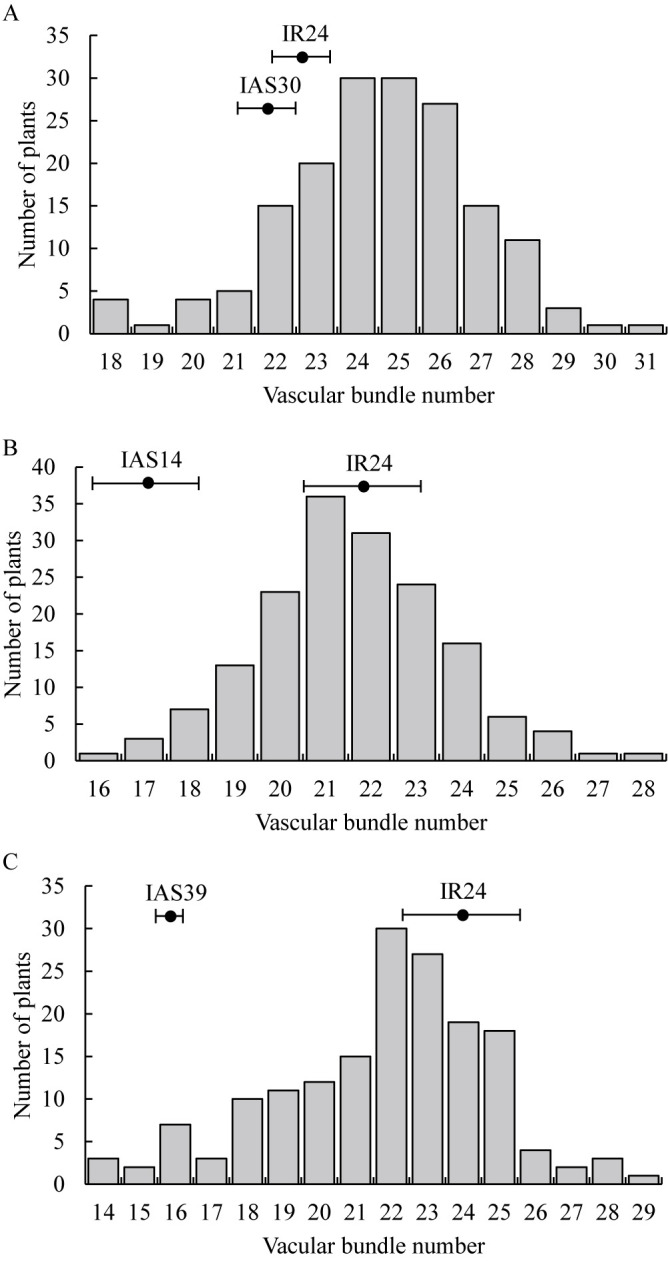
Frequency distributions of vascular bundle number in panicle neck in F_2_ populations derived from (A) IAS30/IR24, (B) IAS14/IR24, (C) IAS39/IR24. Bars indicate means in parents with standard deviation.

VBN of the IR24/IAS30 F_2_ population was 18–31, when that of ‘IR24’ was 23.2 and that of IAS30 was 22.6 (Fig. 3A). Two QTLs were detected on Chr. 5: *qVBN5.1*, with PVE of 12.6%, and *qVBN5.2*, with PVE of 13.7%. The ‘Asominori’ alleles decreased VBN by 1.09 and 1.18, respectively (Table 2). VBN of the IR24/IAS14 F_2_ population was 16–28, when that of ‘IR24’ was 22.2 and that of IAS14 was 17.6 (Fig. 3B). One QTL, *qVBN11*, was detected on Chr. 11, with PVE of 17.9%. The ‘Asominori’ allele decreased VBN by 1.28 (Table 2). VBN of the IR24/IAS39 F_2_ population was 14–29, when that of ‘IR24’ was 24.6 and that of IAS39 was 16.8 (Fig. 3C). One QTL, *qVBN6*, was detected on Chr. 6, with PVE of 61.9%. The ‘Asominori’ allele at *qVBN6* decreased VBN by 3.35 (Table 2). These results confirm the presence of QTLs for VBN, and that the ‘Asominori’ alleles at *qVBN6*, *qVBN5*, and *qVBN11* had negative effects on VBN.

### Evaluation of pyramid effects of two QTLs for VBN in ‘IR24’ genetic background

**Fig. 4.**
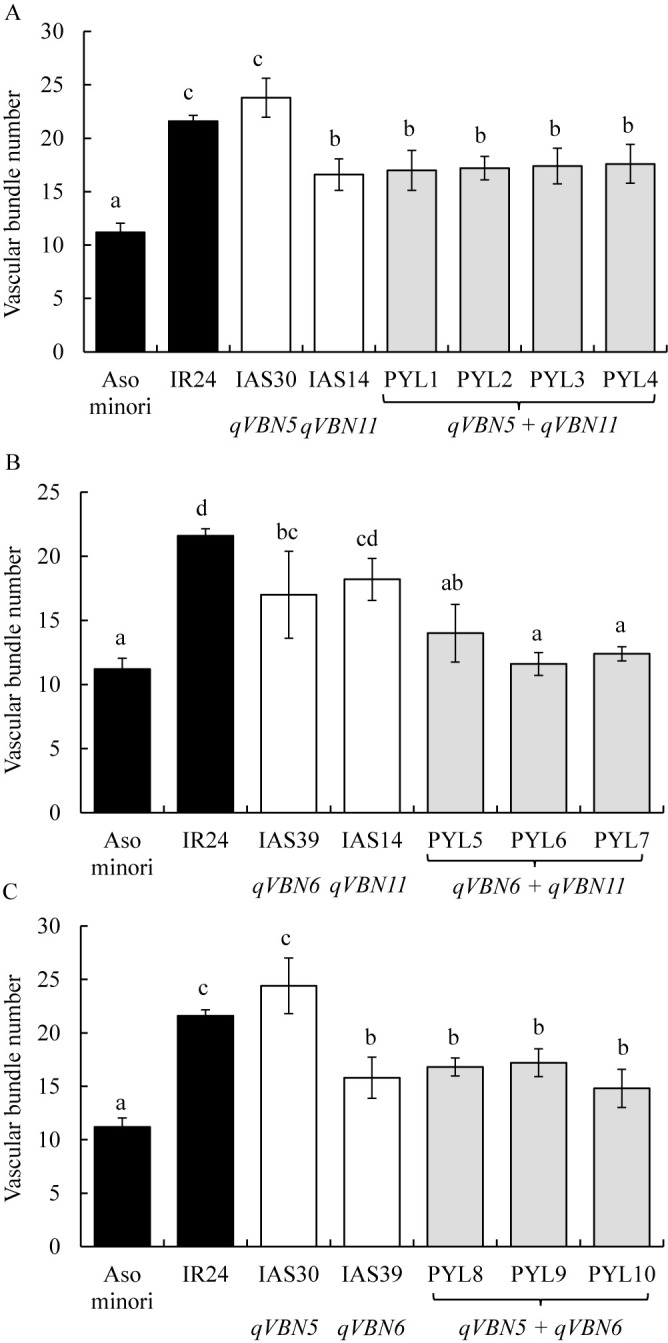
Effects of pyramiding QTLs for vascular bundle number in ‘IR24’ genetic background. (A) *qVBN5* + *11*. (B) *qVBN6* + *11*. (C) *qVBN5* + *6*. Bars with the same letter are not significantly different between genotypes by Tukey–Kramer multiple comparison test (*P* < 0.05).

**Fig. 5.**
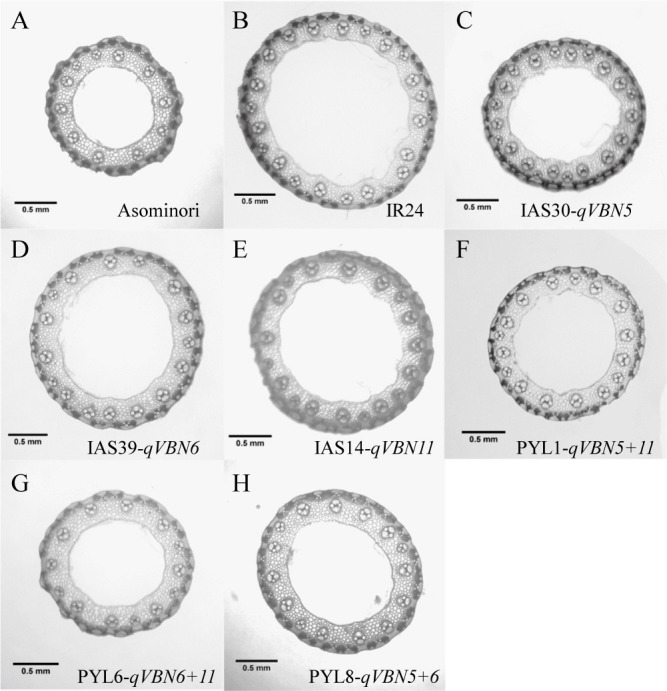
Images of cross-section in the vascular bundle at the panicle neck of parental varieties: (A) ‘Asominori’, (B) ‘IR24’, CSSLs with ‘IR24’ genetic background: (C) IAS30, (D) IAS39, (E) IAS14 and PYLs: (F) PYL1, (G) PYL6, (H) PYL8.

From the IAS30/IAS14 F_2_, PYLs 1–4 (*qVBN5* + *qVBN11*) were selected by markers RM3351, RM6841, C5 Indel8795, and C5 Indel8837. From the IAS39/IAS14 F_2_, PYLs 5–7 (*qVBN6* + *qVBN11*) were selected by markers RM400, RM6395, C5 Indel8795, and C5 Indel8837. And from the IAS30/IAS39 F_2_, PYLs 8–10 (*qVBN5* + *qVBN6*) were selected by markers RM3351, RM6841, RM400, and RM6395. VBNs of PYLs 1–4 (*qVBN5* + *qVBN11*) were 17.0–17.6, similar to that of IAS14 (*qVBN11*) but significantly lower than those of IAS30 (*qVBN5*) and ‘IR24’ (Figs. 4A, 5A–5C, 5E, 5F). VBNs of PYLs 5–7 (*qVBN6* + *qVBN11*) were 11.6–14.0. Those of PYLs 6 and 7 were significantly lower than those of both parental lines, and that of PYL 5 was marginally lower than that of IAS39 (Figs. 4B, 5D, 5E, 5G). VBNs of PYLs 5–7 were not significantly different from that of ‘Asominori’ but were significantly lower than that of ‘IR24’. VBNs of PYLs 8–10 (*qVBN5* + *qVBN6*) were 14.8–17.2, not significantly different from that of IAS39, but significantly lower than those of IAS30 and ‘IR24’ (Figs. 4C, 5C, 5D, 5H).

## Discussion

VBN is a quantitative trait controlled by multiple genes. Many QTLs for VBN in rice have been identified in segregating populations and by GWAS (Bai *et al.* 2012, Cui *et al.* 2003, Liao *et al.* 2021, Sasahara *et al.* 1999, Zhai *et al.* 2018, Zhang *et al.* 2002). RILs and DHs are useful for identification of QTLs with effects across different environments (Collard *et al.* 2005, Kearsey and Farquhar 1998), because environmental influence can be minimized by using multiple replicates over multiple years. Sasahara *et al.* (1999) detected five QTLs for VBN in 3 years’ evaluation by using Asominori × IR24 RILs. They detected one QTL for VBN at 28.64 Mbp on Chr. 6 in all 3 years, one at 26.79 Mbp on Chr. 4 in 2 years, and one at 3.81 Mbp on Chr. 11 and two at 22.56 and 23.29 Mbp on Chr. 5 in 1 year each. Our QTLs correspond to the locations of previously reported QTLs in Sasahara *et al.* (1999) (Table 1) but the locations of QTLs were not exactly overlapped. There were several possibilities for this difference caused by different growing environments and methods of sampling for observing VBN. In Sasahara *et al.* (1999), RILs were grown in Joetsu (37°6ʹ N, 138°15ʹ E) and Niigata (37°55ʹ N, 139°3ʹ E), whereas RILs in this study was grown in Saga (33°14ʹ N, 130°17ʹ E). The temperature during rice growing season were different between these areas and might affect to VBN. Also, 3 panicles were collected from longer tiller in Sasahara *et al.* (1999), while 5 panicles were collected from the tallest tiller among the individual plants in this study. Our number of replications for observed VBN on RILs was higher than that of previous study and it might be affected to different accuracy for QTL detections. In addition, we detected stable QTLs with strong effects and high PVEs: *qVBN5* and *qVBN6* (detected in 3 years) and *qVBN11* (detected in 2 years) had PVE values of 18.3%–28.6%. Sasahara *et al.* (1999) detected two QTLs for VBN on Chrs. 4 and 6 with PVEs of 20%–23%. A significant proportion of QTLs affecting a trait are active across multiple environments, and highly heritable traits are more repeatable and stable across environments (Paterson *et al.* 1991, Tanksley 1993). Thus, *qVBN5*, *qVBN6*, and *qVBN11* were stable owing to their large effects (PVE 18.3%–28.6%) and frequent appearance (2–3 years). Moreover, QTLs for VBN on Chrs. 6 and 11 in the RILs were confirmed as *qVBN6* (27.02–27.65 Mbp) and *qVBN11* (1.18–1.28 Mbp) by using F_2_ populations from IAS39 or IAS14, respectably. Meanwhile two QTLs for *qVBN5*, *qVBN5.1* and *qVBN5.2*, around 21.11–22.52 and 24.44–24.82 Mbp, respectively (Table 2) were close to the regions of two QTLs detected by Sasahara *et al.* (1999) around 22.56 and 23.29 Mbp. These results suggest that CSSLs have higher sensitivity for QTL detection and can accurately evaluate effects of a single QTL. These results will contribute to knowledge of the complex genetics of VBN in the panicle neck in rice and set the foundation for potential fine mapping and cloning of these loci.

The phenotypic variation of each QTL detected in the RILs differed by year. *qVBN5* and *qVBN6* appeared in all 3 years, with PVEs of 10.3%–28.6% and 13.6%–18.3%, respectively; *qVBN11* appeared in 2 years, with PVEs of 11.8%–24.0%; and *qVBN4* appeared only in 2018, with PVE of 10.4% (Table 1). The total PVE of the three QTLs detected in each year (44.4% in 2016, 66.2% in 2017, and 39.0% in 2018) cannot explain all of the difference in VBN between the parents. Other genetic factors might also control VBN. In addition, the differences in results between our study and Sasahara *et al.* (1999) might be due to testing of RILs derived from the same parents in different environments, implying the influence of environmental factors on VBN and the error of collecting the panicle. In this study, ‘IR24’ had around 15 panicles and each panicle in one plant had slightly different VBN. Therefore, to prevent the error of collecting panicle, we selected thicker panicle neck and tallest panicle in one plant because the VBN was correlated to panicle thickness (Lee *et al.* 1992). Thus, we could identify three QTLs for VBN using RILs in multiple years and three QTLs were confirmed in F_2_ populations.

The QTLs for VBN on Chrs. 5 and 11 only detected in segregating populations derived from a cross between ‘Asominori’ and ‘IR24’. In contrast, QTLs on Chrs. 4 and 6 were located near QTLs for VBN reported previously: on Chr. 4 at 31.07–31.26 Mbp (Zhai *et al.* 2018) and at 22.0–26.8 Mbp (Cui *et al.* 2003). Here, *qVBN4* was located around 24.7 Mbp, near *NAL1* (Os04g0615000) at around 31.2 Mbp on Chr. 4. *NAL1* controls the leaf vein pattern and increases panicle neck diameter and VBN (Fujita *et al.* 2013, Qi *et al.* 2008). *qVBN6* was located around 28.6 Mbp, near *APO1* (Os06g0665400) at around 27.46 Mbp on Chr. 6. *APO1* enhances the development of vascular bundles, promoting carbohydrate translocation to the panicle (Terao *et al.* 2010) and therefore they might house the same gene.

The phenotypic effects of QTLs cannot be precisely confirmed through the use of RILs (Yamamoto *et al.* 2009), but can be determined through the use of CSSLs. As the genotypes of CSSLs are uniform and phenotypic variation is due mainly to the substitution segments carrying target QTLs, each QTL detected in the RILs could be validated after the evaluation of CSSLs in two years. VBNs showed similar tendencies between years, and only IAS14 and IAS39 not IAS30 were lower than that of ‘IR24’ (Fig. 2), implying a stable effect of these QTLs. The VBN of IAS39 carrying *qVBN6* decreased from 6.6 to 8.1 compared to that of ‘IR24’. The VBN of IAS14 carrying *qVBN11* decreased from 3.3 to 4.9 compared to that of ‘IR24’. The genetic background of each CSSL is ~93% similar to ‘IR24’ (Kubo *et al.* 2002). Thus, the reductions of VBN in each CSSL were caused by the presence of the ‘Asominori’ chromosomal segment in the target region of each detected QTL. Additionally, the substituted ‘Asominori’ segment still present in the CSSL (*i.e.* IAS30 carrying ‘Asominori’ segment on Chrs. 1 and 11, IAS14 carrying ‘Asominori’ segment on Chr. 3) may also relate to a decrease in VBN in each CSSL. On the other hand, the effect of *qVBN5* on IAS30 (carrying *qVBN5*) was similar to that of ‘IR24’. IAS30 was taller and had a thicker culm than ‘IR24’, to which we attribute the higher VBN. Lee *et al.* (1992) found a significant correlation between VBN, panicle length, and internode thickness. Therefore, peduncles on taller plants are thicker, and the plants have a higher VBN.

To understand the interactions of QTLs related to VBN, we developed pyramided lines carrying pairs of QTLs. The VBNs of PYLs 1–4 (*qVBN5* + *qVBN11*) and of PYLs 8–10 (*qVBN5* + *qVBN6*) were the same as those of the parental lines IAS14 and IAS39, respectively. Based on our observations, plants of PYLs 1–4 and PYLs 8–10 were taller than ‘IR24’ owing to inheritance of the tall plant type from IAS30 (carrying *qVBN5*). Therefore, tall plant type might influence peduncle thickness and VBN. The VBNs of PYLs 5–7 (*qVBN6* + *qVBN11*) were significantly less than those of both parental lines, so the effects of the two QTLs on VBN might be independent in the ‘IR24’ genetic background. In addition, the VBN of PYL 6 was 11.6, close to that of ‘Asominori’ (11.2 VBN), whereas PYL 7 differed from those of both IAS39 and IAS14. Thus, the interaction between *qVBN6* and *qVBN11* is additive effect. *qVBN6* and *qVBN11* decreased respectively by 6.6–8.1 and 3.3–4.9 VBN in IAS39 and IAS14, and both by 10 VBN in PYL6. In several previous study, the interactions between QTLs for morphological traits such as panicle architecture, grain length, grain width has been identified as additive effect (Ando *et al.* 2008, Liang *et al.* 2021). The pyramiding effects of ‘Habataki’ alleles on *qSBN1*, which controlled secondary branch number, and *qPBN6*, which controlled primary branch number showed an additive increase in spikelet number per panicle (Ando *et al.* 2008). The pyramiding effects of Z563 alleles on *qGL3-1*, *qGL3-2*, and *qGL7*, which controlled grain length, resulted in an additive decrease in grain length, whereas pyramiding effects of *qGW3-1* and *qGW3-2* which controlled grain width were additive increase in the grain width (Liang *et al.* 2021). There were no previous studies for revealing pyramiding effects on QTLs for VBN. In our study, the interaction of *qVBN6* and *qVBN11* was found to be additive to decrease in VBN. The additive interaction between QTLs for VBN was similar to previous studies for interaction of QTLs for morphological traits.

In this study, we confirmed and validated the detected QTLs in ‘Asominori’ and ‘IR24’ genetic background. In CSSLs with ‘IR24’ genetic background: IAS14, IAS30 and IAS39, the effect of ‘Asominori’ allele on QTLs decreased by 1–7 VBN. However, the effect of ‘IR24’ allele on QTLs increased by only 1–2 VBN in CSSLs with ‘Asominori’ genetic background: AIS38, AIS49 and AIS76 (Supplemental Table 2). The effect of QTLs on VBN in the ‘IR24’ genetic background was larger than that in the ‘Asominori’ genetic background. In the F_2_ IAS30/IR24 for *qVBN5* and IAS14/IR24 for *qVBN11*, QTLs for VBN were detected and showed phenotypic variation with 12.6%–13.7% and 17.9%, respectively (Table 2). In the F_2_ AIS38/Asominori for *qVBN5* and AIS76/Asominori for *qVBN11*, QTLs for VBN were detected but showed less phenotypic variation with 8.1% and 7.5%, respectively (Supplemental Table 3). Also, the phenotypic variance of QTLs on VBN in the ‘IR24’ genetic background was larger than that in the ‘Asominori’ genetic background. The effects of QTLs for VBN were different between ‘Asominori’ and ‘IR24’ genetic background.

Previously, there are several studies found that there was a significant correlation between the VBN on the panicle neck and panicle structure in segregating populations of the *indica* and *japonica* rice cross (Fukuyama and Takayama 1995, Liao *et al.* 2021, Sasahara *et al.* 1999, Zhai *et al.* 2018). In our study, CSSLs carrying a single QTL for VBN with ‘Asominori’ genetic background increased VBN comparing with ‘Asominori’ and also influenced panicle architecture: the primary branch number (PBN), secondary branch number (SBN), and total spikelet numbers (TSN) (Supplemental Table 4). The PBN, SBN and TSN on AIS49 carrying *qVBN6* had significantly higher than on ‘Asominori’ in two years. PBN and TSN on AIS38 carrying *qVBN5* and SBN and TSN on AIS76 carrying *qVBN11* were significantly higher than that on ‘Asominori’ in 1 or 2 years. With increasing VBN, these QTLs increased branch and spikelet number on panicles. Terao *et al.* (2010) reported that ‘Habataki’ allele on *APO1* increased PBN and VBN that enhanced carbohydrate translocation to panicles, resulting in higher grain yield per plant. The effects of ‘IR24’ allele on *qVBN5*, *qVBN6*, and *qVBN11* tended to increase TSN and VBN, as well as ‘Habataki’ allele on *APO1*. Based on these results, there is possibility that ‘IR24’ allele on *qVBN5*, *qVBN6*, and *qVBN11* would increase grain yield in *japonica* varieties by increasing branch and spikelet number and VBN. However, to confirm the effects of these QTLs in detailed, it is necessary to evaluate yield and yield components in future study. Once the location of these QTLs is narrowed down by fine mapping, the QTLs for VBN could be introduced into other *japonica* varieties and in developing *indica-japonica* cross cultivars as well as higher yield varieties to improve the yield trait through MAS. In addition, one or two QTLs might not be sufficient to increase VBN in the ‘Asominori’ genetic background. Therefore, finding several QTLs for VBN with minor effects will be necessary to develop *japonica* varieties with high VBN.

## Author Contribution Statement

All authors contributed to the study conception and design. Material preparation, data collection and analysis were performed by HTLN, SS, YN and ZD. The manuscript was written by HTLN, SHZ and DF.

## Supplementary Material

Supplemental Tables

## Figures and Tables

**Table 1.** Detection of QTLs for vascular bundle number in RILs derived from IR24/Asominori

Year	QTL	Chr.	Interval marker	LOD	Additive effect*^a^*	*R*^2^ (%)
2016	*qVBN5*	5	C128–R2117	4.6	–1.27	16.3
	*qVBN6*	6	C962–Ky11	4.2	–1.31	16.3
	*qVBN11*	11	R2918–C3029A	3.4	–1.14	11.8
2017	*qVBN5*	5	C128–R2117	6.9	–1.67	28.6
	*qVBN6*	6	Xnpb135–C962	3.8	–1.21	13.6
	*qVBN11*	11	C794A–C83B	6.3	–2.19	24.0
2018	*qVBN4*	4	C335–C621B	3.5	0.84	10.4
	*qVBN5*	5	C128–R2117	3.4	–0.86	10.3
	*qVBN6*	6	C962–Ky11	5.2	–1.15	18.3

*^a^* Additive effects indicate ‘Asominori’ allele.

**Table 2.** Detection of QTLs on Chrs. 5, 6, and 11 in CSSLs with ‘IR24’ background

QTL	Chr.	Interval marker	Interval marker (Mbp)*^a^*	LOD	Additive effect*^b^*	Dominant effect	PVE (%)
*qVBN5.1*	5	RM18751–RM18821	21.11–22.52	4.6	–1.09	0.54	12.6
*qVBN5.2*	5	RM7081–RM7446	24.44–24.82	5.0	–1.18	0.75	13.7
*qVBN6*	6	RM20546–Indel493	27.02–27.65	34.5	–3.35	–1.45	61.9
*qVBN11*	11	Indel8807–Indel8810	1.18–1.28	7.0	–1.28	0.02	17.9

*^a^* On ‘Nipponbare’ genome sequence. *^b^* Negative sign indicates negative-effect ‘Asominori’ allele.
