# Supplementary material for: Identification and characterization of stable QTLs for vascular bundle number at the panicle neck in rice (Oryza sativa L.)
Source: Breed Sci. 2023 Sep 9;73(4):365–72. doi: 10.1270/jsbbs.23013 (PMC10722095; doi:10.1270/jsbbs.23013)
Supplement: Supplementary file 1 — Supplemental Tables [file 73_365_s1.pdf]

doi: 10.1270/jsbbs.23013

Supplemental Table 1. The SSR and indel markers for maker-assisted selection of three Chrs. 5, 6 and 11

Marker[a]	Chr.	Forward primer sequence (5' -> 3')	Reverse primer sequence (5' -> 3')	Physical position (Mbp)[b]	Predicted size (bp)[c]
RM3351	5	GTCGAAACGTAGCCAGGCAATGG	CCATGGAAGGAATGGAGGTGAGG	20.62	139
RM18727	5	GGTGGTCACGCTTCTTCTCCTCTCC	CGGCGGTGTGATTCTCCTCTCC	20.76	99
RM18751	5	CCGTGTGTTGGCTTAGAATCAAGG	GCCACTTTCCAAACATCAGAAAGC	21.11	150
RM18821	5	CGGCTAAATCGTCATGTGTATGG	TCTCCCATCTTACATGTCCTCACC	22.52	186
RM6841	5	CTTCCCGAAATCAGATTCTTGC	CGACGAGTCCTACACACTCTCC	22.66	576
RM6054	5	AGGCTCTTCGGCTTCATCTCC	GGTCTCTGATCAGTTTGCTTTGG	22.7	190
KNJ8-indel384	5	ACGTCAGCGCCTGATTTC	TATTCTGTGTTCGAGCCATGTG	23.47	145
KNJ8-indel385	5	GCCACGTGGACTATAGAGGAGA	CCCAAGTCAATCCAATAGTTTCTT	23.52	142
RM18910	5	CACCCAATATGAGTACGGAACAGC	ATACTTGCCGGTATCTTCGTTCC	23.98	153
RM18914	5	GCTCTCCATGTGTTATCAGCAACC	GAGAAAGAGTTTCTTCTGCCTGTTGG	24.04	299
RM18926	5	CTACCTCTCCGTCCGCATCC	AAGAGGGACTCAGAGAGCACTTCC	24.34	249
RM7081	5	CTTCCCGCACTACACTGCACTCC	CTGCAACTTGCTCATGGAGTTGG	24.44	97
RM7446	5	CGTTGAGCCAAGAAGAAGAAAGG	TTTGAAGGCAGTTTCACTGACG	24.82	195
RM3348	5	CTTCTCGGTTCATCCAAAGAGC	GTGGAAGCTATGGGTAGCTCACG	24.95	88
RM3170	5	GCAGTGTCATTCTCATGAAACCTACC	CAGACTCCAAAGCACCCATAACC	27.8	180
RM6395	6	GGCTTCGGCTTCTGAACTAGC	CGACTAAGCAGCAGTAACAATCTCG	25.61	93
RM20546	6	TGAGCAGGAGACGGGACAGC	TATCCGTTTCTGCAACGCTACGC	27.02	170
KNJ8-indel493	6	TTTCTGCTCCTGAAACACGTTA	GCAGCACAAAGCTCTATCTATCA	27.65	149
RM20596	6	AACTTCCTTTCCAGGCTTTCAGC	TTCACTGAGCCTGAACACATTGC	27.71	170
RM400	6	TTACACCAGGCTACCCAAACTCG	TTGCTGAGTTCCCTCGTCTATCC	28.05	380
RM3138	6	GTGGTGAATGTTGAGCTGCATGG	GACTGAGCCAAGTTGCTGTCTGG	28.09	199
RM3343	6	ATCAAGGCGGTACAGGCACACG	CGCAGATGGCTGAAACCCTAACC	29.1	250

C5-indel8784	11	CGACAAGTGATGCGACAATAAT	GTAACACAACGAAGGAGCAACA	0.68	138
RM3225	11	GATAGAGGATTGGGTGCGTGTGC	TACGCCAACCAATTCCAAACACC	0.89	177
RM3863	11	CGATTGATCCCGTGCAAGTAGG	CATGCTAGTGCATTCTGCGTAGG	0.9	147
C5-indel8802	11	GATGTAAGAAGCCAGGCACAC	GACGTGGCAATATTCTTTGACA	1.09	87
C5-indel8807	11	ACAACGACAAACAGTACGATGC	GCGCATTCACCTTCAGTAACTT	1.18	96
C5-indel8810	11	ACAACTCCAGGAGAGCCAGATA	TTAGAATCCAACGCATCCTCTT	1.28	118
C5-indel8828	11	CCGGAAACAGTCATGGTACATT	GATGGGAATCGGAACTATTTGA	1.55	122
C5-indel8833	11	AAGGGCACAAACCAATAATTAGA	GCACTTTCCGATCAGTTAAACA	1.67	122
C5-indel8834	11	CTGCAAGCTCCACTTCACTG	AGGGCGAAGGAGATGGAC	1.8	97
RM26045	11	GAGCTTACCTTCACCAAGTAGACC	GTAGAACATCTACCATCGAGTGC	1.9	298
RM26051	11	ATCAGCATATCTCCCTGCAAAGC	GGTTCTTCACCCGCCATATTCC	1.99	182
RM26076	11	CCTTCTCTCTCCCACATCTCTAGC	CTAGTGAGTCCCACGTGTCAACC	2.48	238
C5-indel8795*	11	GAACCAATGTTGAGATGTTTCC	GAAAGCAATTTCGCTTATCTCAA	0.86	139
C5-indel8837*	11	GAACATGCAAGGACCATACTAAA	GGGTAGTGCTTGTTTATTTCAA	1.83	150

[a] Markers with RM were from McCouch *et al.* 2002, while KNJ8- and C5- indel were from Yonemaru *et al.* 2015

[b] The marker's physical position was determined by the forward primer's location, obtained from The Rice Annotation Project Database

[c] The predicted size is determined by the reference genome ('Nipponbare' IRGSP v.1.0).

[*] The markers used for selecting *qVBN11*.

Supplemental Table 2. Validation of single QTL effects on VBN in' Asominori' genetic background

Line		2017	2018	2019	2019KU
Asominori		11.8 ± 0.5	10.4 ± 0.69	11.5 ± 0.85	9.6 ± 0.89
AIS38	*qVBN5*	11.6 ± 1.1	11.2 ± 0.42 *	12.2 ± 1.03	11.2 ± 0.84
AIS49	*qVBN6*	13.0 ± 1.2	12.2 ± 0.78 ***	13.5 ± 0.53 ***	12.4 ± 1.14 **
AIS76	*qVBN11*	12.5 ± 1.7	11.5 ± 0.71 **	12.8 ± 0.92 **	10.8 ± 1.09

2019KU is evaluated at Kyushu University

Dunnett’s test was conducted and significant level at 5 %, 1 % and 0.1 % indicated *, ** and ***, respectively

Supplemental Table 3. Detection of QTLs for VBN in F_2 populations from CSSLs with 'Asominori' genetic background

QTL	Chr.	Interval marker	Interval marker (Mbp)[a]	Additive effect [b]	LOD[c]	Dominant effect	PVE (%)
qVBN5	5	RM3351–RM6841	20.62 - 22.66	0.54	2.45	-0.24	8.1
qVBN11	11	Indel8802–Indel8807	1.18–1.28	0.46	2.77	-0.01	7.5

[a] The physical distance was based on 'Nipponbare' genome sequence.

[b] Positive sign indicates negative-effect 'IR24' allele

[c] Threshold of LOD was 1.8 in AIS38/Asominori F_2 population and 1.7 in AIS76/Asominori F_2 population by permutation test with significant level of $P < 0.05$

Supplemental Table 4. Validation of single QTL effects on panicle-related trait in' Asominori' genetic background

CSSL	QTL	PBN			SBN			TSN		
		2018	2019	2019K	2018	2019	2019K	2018	2019	2019K
AIS38	*qVBN5*	11.0 ± 0.4 **	10.1 ± 1.4	9.8 ± 0.4	16.8 ± 3.2	18.4 ± 2.5	16.2 ± 2.9	108.8 ± 9.6 **	104.2 ± 10.3	100.0 ± 5.7 *
AIS49	*qVBN6*	12.3 ± 0.8 ***	13.5 ± 0.5 ***	13.0 ± 0.7 ***	21.0 ± 3.8 ***	27.8 ± 4.5 ***	31.0 ± 3.4 ***	120.4 ± 12.1 ***	144.8 ± 14.5 ***	152.0 ± 7.2 ***
AIS76	*qVBN11*	9.9 ± 0.8	11.1 ± 1.0	9.6 ± 1.1	18.8 ± 2.2 **	21.7 ± 1.4	22.0 ± 3.4 ***	103.9 ± 7.1	121.0 ± 7.4	112.4 ± 16.3 ***
Asominori	Asominori	9.9 ± 0.3	10.6 ± 1.1	9.0 ± 1.0	14.1 ± 1.3	18.7 ± 3.8	13.0 ± 2.1	94.4 ± 6.6	109.2 ± 13.4	82.0 ± 7.7

PBN: primary branch number, SBN: secondary branch number, TSN: total spikelet numbers per panicle

Dunnett’s test was conducted and significant level at 5 %, 1 % and 0.1 % indicated *, ** and ***, respectively
